# *Mycobacterium tuberculosis* PhoY Proteins Promote Persister Formation by Mediating Pst/SenX3-RegX3 Phosphate Sensing

**DOI:** 10.1128/mBio.00494-17

**Published:** 2017-07-11

**Authors:** Sarah B. Namugenyi, Alisha M. Aagesen, Sarah R. Elliott, Anna D. Tischler

**Affiliations:** Department of Microbiology and Immunology, University of Minnesota, Minneapolis, Minnesota, USA; Washington University in St. Louis School of Medicine

**Keywords:** *Mycobacterium tuberculosis*, PhoU, Pst system, RegX3, antibiotic tolerance, persister, phosphate, rifampin

## Abstract

The *Mycobacterium tuberculosis* phosphate-specific transport (Pst) system controls gene expression in response to phosphate availability by inhibiting the activation of the SenX3-RegX3 two-component system under phosphate-rich conditions, but the mechanism of communication between these systems is unknown. In *Escherichia coli*, inhibition of the two-component system PhoR-PhoB under phosphate-rich conditions requires both the Pst system and PhoU, a putative adaptor protein. *E. coli* PhoU is also involved in the formation of persisters, a subpopulation of phenotypically antibiotic-tolerant bacteria. *M. tuberculosis* encodes two PhoU orthologs, PhoY1 and PhoY2. We generated *phoY* single- and double-deletion mutants and examined the expression of RegX3-regulated genes by quantitative reverse transcription-PCR (qRT-PCR). Gene expression was increased only in the Δ*phoY1* Δ*phoY2* double mutant and could be restored to the wild-type level by complementation with either *phoY1* or *phoY2* or by deletion of *regX3*. These data suggest that the PhoY proteins function redundantly to inhibit SenX3-RegX3 activation. We analyzed the frequencies of antibiotic-tolerant persister variants in the *phoY* mutants using several antibiotic combinations. Persister frequency was decreased at least 40-fold in the Δ*phoY1* Δ*phoY2* mutant compared to the frequency in the wild type, and this phenotype was RegX3 dependent. A Δ*pstA1* mutant lacking a Pst system transmembrane component exhibited a similar RegX3-dependent decrease in persister frequency. In aerosol-infected mice, the Δ*phoY1* Δ*phoY2* and Δ*pstA1* mutants were more susceptible to treatment with rifampin but not isoniazid. Our data demonstrate that disrupting phosphate sensing mediated by the PhoY proteins and the Pst system enhances the susceptibility of *M. tuberculosis* to antibiotics both *in vitro* and during infection.

## IMPORTANCE

Persister variants, subpopulations of bacteria that are phenotypically antibiotic tolerant, contribute to the lengthy treatment times required to cure *Mycobacterium tuberculosis* infection, but the molecular mechanisms governing their formation and maintenance are poorly characterized. Here, we demonstrate that a phosphate-sensing signal transduction system, comprising the Pst phosphate transporter, the two-component system SenX3-RegX3, and functionally redundant PhoY proteins that mediate signaling between Pst and SenX3-RegX3, influences persister formation. Activation of RegX3 by deletion of the *phoY* genes or a Pst system component resulted in decreased persister formation *in vitro*. Activated RegX3 also limited persister formation during growth under phosphate-limiting conditions. Importantly, increased susceptibility to the front-line drug rifampin was also observed in a mouse infection model. Thus, the *M. tuberculosis* phosphate-sensing signal transduction system contributes to antibiotic tolerance and is a potential target for the development of novel therapeutics that may shorten the duration of tuberculosis treatment.

## INTRODUCTION

In 2015, there were an estimated 10.4 million new cases of active tuberculosis (TB) infection caused by *Mycobacterium tuberculosis* worldwide and approximately 1.8 million deaths attributed to the infection (1). The standard treatment for TB is a 6- to 9-month multidrug regimen consisting of isoniazid, rifampin, ethambutol, and pyrazinamide. The long duration of treatment often leads to patient noncompliance, a factor that has contributed to the rise of drug-resistant *M. tuberculosis* strains (2, 3). One feature of *M. tuberculosis* that may contribute to the long-term therapy required to cure infections is its ability to form persisters, a subpopulation of bacterial cells that are phenotypically tolerant to antibiotics but genetically identical to drug-susceptible bacteria (4–6). Determining the underlying mechanisms by which *M. tuberculosis* forms persisters is important because targeting these pathways could shorten TB treatment.

Although persisters do not possess the stable and heritable genetic mutations that characterize antibiotic resistance, genetic factors can influence persister frequency. *Escherichia coli* has served as a model for identifying mechanisms of persister formation (7). *E. coli phoU* was identified as a persister gene in a transposon mutagenesis screen; the *phoU* mutant had reduced persister frequency in cultures exposed to ampicillin (8). PhoU has two known functions. It regulates the uptake of inorganic phosphate (P_i_) by the phosphate-specific transport (Pst) system, a high-affinity ATP-binding cassette (ABC)-type transporter that scavenges P_i_ during P_i_-limited conditions (9). PhoU and the Pst system also participate in a signal transduction system that senses environmental P_i_ to regulate the expression of genes involved in P_i_ homoeostasis and, in the case of pathogens, virulence (10). When P_i_ is in excess, the Pst system inhibits the activation of the two-component regulatory system PhoR-PhoB. When P_i_ becomes limiting, this inhibition is relieved, the DNA binding response regulator PhoB is activated, and the P_i_-responsive Pho regulon is transcribed (10). PhoU is also required to inhibit the activation of PhoR-PhoB under P_i_-rich conditions (11), possibly via direct physical interactions with the Pst cytoplasmic ATPase subunit PstB and the PhoR sensor histidine kinase (12). However, the mechanism by which PhoU promotes the formation of antibiotic-tolerant persister variants in *E. coli* remains unknown.

Mycobacteria use a similar two-component regulatory system, SenX3-RegX3, to sense and respond to P_i_ limitation (13, 14) and nutrient starvation (15). In *M. tuberculosis*, SenX3-RegX3 activity is controlled in response to extracellular P_i_ by a Pst P_i_ transport system (16). The deletion of *pstA1*, which encodes a Pst system transmembrane component, resulted in aberrant expression of P_i_-responsive genes under P_i_-rich conditions, hypersensitivity to *in vitro* stress conditions, and sensitivity to host immune responses *in vivo* (16). These Δ*pstA1* mutant phenotypes were attributed to constitutive activation of SenX3-RegX3 (16), suggesting that the *M. tuberculosis* Pst system controls the expression of P_i_-responsive genes by inhibiting the activation of SenX3-RegX3 under P_i_-rich conditions. *M. tuberculosis* requires the ability to sense and respond to fluctuating P_i_ availability via the Pst/SenX3-RegX3 signal transduction system for virulence (16, 17), but the mechanism by which the Pst system controls the activity of SenX3-RegX3 has not been determined.

*M. tuberculosis* encodes two putative PhoU orthologs, PhoY1 and PhoY2. It is unknown whether these proteins participate in P_i_ signaling, but some evidence suggests that PhoY2 promotes the formation of antibiotic-tolerant persisters (18). An *M. tuberculosis* Δ*phoY2* mutant exhibited lower persister frequency after exposure of stationary-phase cultures to either pyrazinamide or rifampin and failed to persist in the lungs and spleens of infected mice (18). In *Mycobacterium marinum*, a pathogenic relative of *M. tuberculosis*, a *phoY2* transposon mutant was hypersusceptible to several antimycobacterial antibiotics, nutrient starvation, and cell wall stress (19). Although these data suggest that mycobacterial PhoY2 is required for persister formation and survival of mycobacteria under stress conditions, our preliminary experiments suggested that the PhoY proteins function redundantly to control the activation of RegX3 in *M. tuberculosis*.

We therefore hypothesized that both PhoY proteins facilitate communication between the Pst system and SenX3-RegX3 and that disrupting this P_i_-sensing signal transduction would enhance the susceptibility of *M. tuberculosis* to antibiotics. Here, we show that deletion of both *phoY1* and *phoY2* is required for significant dysregulation of RegX3-dependent P_i_-responsive genes and sensitivity to stress during *in vitro* growth under P_i_-rich conditions. This suggests functional redundancy of PhoY1 and PhoY2 in mediating the response of *M. tuberculosis* to environmental P_i_ availability. Additionally, we demonstrate reduced persister frequency *in vitro* for both Δ*phoY1* Δ*phoY2* and Δ*pstA1* mutants that is *regX3* dependent. Both the Δ*phoY1* Δ*phoY2* and Δ*pstA1* mutants are also more efficiently cleared from infected mice due to the combined effect of host immune responses and antibiotic treatment. Our results suggest that the *M. tuberculosis* PhoY proteins promote persister formation both *in vitro* and during infection by preventing activation of the *M. tuberculosis* SenX3-RegX3 P_i_-responsive signal transduction pathway.

## RESULTS

### PhoY1 and PhoY2 function redundantly to inhibit RegX3-dependent gene expression.

**FIG 1:**
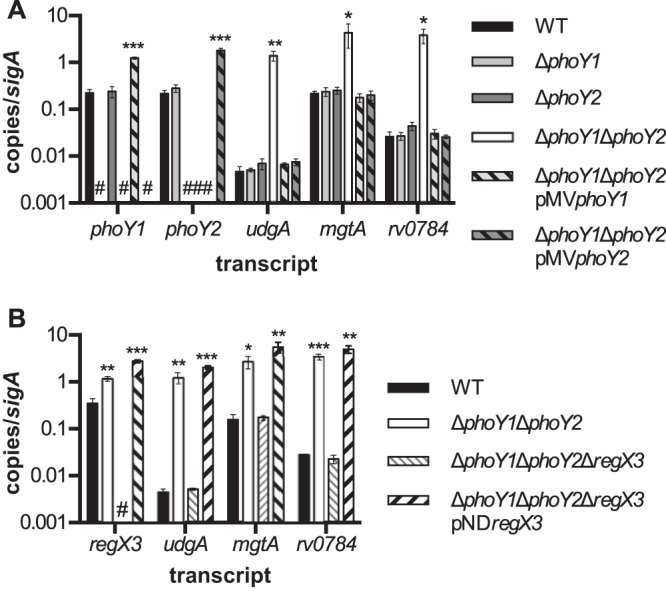
RegX3-regulated genes are overexpressed in the Δ*phoY1* Δ*phoY2* mutant. RNA was isolated from *M. tuberculosis* WT, and the indicated deletion mutants grown to mid-exponential phase (OD_600_ 0.5) in 7H9 medium. Expression of select transcripts was measured using quantitative reverse transcription-PCR, and the results normalized to the *sigA* transcript level. Data shown are the mean values ± standard deviations of three independent experiments. #, no detectable transcript. Asterisks indicate statistically significant transcript levels compared to the results for the WT, as follows: *, *P* < 0.05; **, *P* < 0.005; ***, *P* < 0.0005. (A) *phoY1*, *phoY2*, *udgA*, *mgtA*, and *rv0784* transcripts were measured in *M. tuberculosis* WT and Δ*phoY1*, Δ*phoY2*, Δ*phoY1* Δ*phoY2*, Δ*phoY1* Δ*phoY2/*pMV*phoY1*, and Δ*phoY1* Δ*phoY2/*pMV*phoY2* mutants. (B) *regX3*, *udgA*, *mgtA*, and *rv0784* transcripts were quantified in *M. tuberculosis* WT and Δ*phoY1* Δ*phoY2*, Δ*phoY1* Δ*phoY2* Δ*regX3*, and Δ*phoY1* Δ*phoY2* Δ*regX3*/pND*regX3* mutants.

PhoY1 and PhoY2 are 63% identical (80% similar) and are 40% and 44% similar, respectively, to *E. coli* PhoU. To determine whether *M. tuberculosis* PhoY1 and/or PhoY2 limits P_i_-responsive gene expression when P_i_ is abundant, similarly to *E. coli* PhoU, we constructed mutants with in-frame unmarked deletions of both genes in the Erdman strain. Single Δ*phoY1* and Δ*phoY2* deletion mutants and a double Δ*phoY1* Δ*phoY2* mutant were made and validated by Southern blotting (see Fig. S1 in the supplemental material). Furthermore, the *phoY1* and *phoY2* transcripts were not detectable in the Δ*phoY1* and Δ*phoY2* mutants, respectively, by quantitative reverse transcription-PCR (qRT-PCR) (Fig. 1A). Neither *phoY* transcript was detected in the Δ*phoY1* Δ*phoY2* mutant (Fig. 1A).

10.1128/mBio.00494-17.1 FIG S1  Confirmation of *phoY1* and *phoY2* chromosomal deletions by Southern blotting. (A and B) Genomic DNA from *M. tuberculosis* Erdman (WT) and Δ*phoY1*, Δ*phoY2*, and Δ*phoY1* Δ*phoY2* mutants was digested with the indicated restriction enzymes, and bands that hybridized to a gene-specific probe were detected by chemiluminescence. Positions of molecular size markers are indicated. (A) PstI digest and *phoY1* probe that hybridized to a 1.8-kbp fragment from the WT and a 1.1-kbp fragment from the Δ*phoY1* mutants. (B) XhoI digest and *phoY2* probe that hybridized to a 2.9-kbp fragment from the WT and a 2.3-kbp fragment from the Δ*phoY2* mutants. (C and D) Maps of the *phoY1* (C) and *phoY2* (D) loci. Genes are indicted by gray arrows. Probes are indicated by red bars. Download FIG S1, EPS file, 0.7 MB. Copyright © 2017 Namugenyi et al. 2017 Namugenyi et al. This content is distributed under the terms of the Creative Commons Attribution 4.0 International license.

We predicted that *M. tuberculosis* PhoY1 and/or PhoY2 would participate in P_i_ sensing with the Pst/SenX3-RegX3 signal transduction system. We previously identified many genes that were significantly overexpressed by Δ*pstA1* mutant bacteria during growth under P_i_-rich conditions, including *udgA*, *mgtA*, and *rv0784* (16). Overexpression of these genes was dependent on the DNA binding response regulator RegX3 (16), though it remains unknown whether this regulation is direct or indirect. We examined the expression of these three genes in the *phoY* deletion mutants using qRT-PCR. The expression levels of *udgA*, *mgtA*, and *rv0784* were unchanged in both the Δ*phoY1* and the Δ*phoY2* single mutant (Fig. 1A). In Δ*phoY1* Δ*phoY2* bacteria, however, each gene was significantly overexpressed compared to its expression in the wild-type (WT) control (Fig. 1A). To verify that the *phoY1* or *phoY2* deletion caused these changes in gene expression, we complemented the Δ*phoY1* Δ*phoY2* mutant by providing either *phoY1* or *phoY2* under the control of its native promoter in *trans* on the episomal plasmid pMV261. Complementation with either *phoY1* or *phoY2* restored the expression of *udgA*, *mgtA*, and *rv0784* to WT levels despite significant overexpression of *phoY1* and *phoY2* from the complementing plasmids (Fig. 1A). These results indicate that PhoY1 and PhoY2 function redundantly to inhibit gene expression during growth under P_i_-rich conditions.

To determine if aberrant gene expression in the Δ*phoY1* Δ*phoY2* mutant is dependent on RegX3, we constructed an in-frame unmarked deletion of *regX3* in the Δ*phoY1* Δ*phoY2* mutant. The *regX3* transcript was undetectable in Δ*phoY1* Δ*phoY2* Δ*regX3* bacteria, confirming deletion of *regX3* (Fig. 1B). *udgA*, *mgtA*, and *rv0784* were each expressed at the WT level in Δ*phoY1* Δ*phoY2* Δ*regX3* bacteria, suggesting that these genes are overexpressed in the double *phoY* mutant due to constitutive activation of RegX3 (Fig. 1B). Complementation of Δ*phoY1* Δ*phoY2* Δ*regX3* bacteria with pND*regX3*, encoding *regX3* under the control of its native promoter on an integrating vector, resulted in overexpression of the *udgA*, *mgtA*, and *rv0784* transcripts at levels comparable to those in the Δ*phoY1* Δ*phoY2* mutant (Fig. 1B). These results indicate that PhoY1 and PhoY2 inhibit the activation of RegX3 under P_i_-rich conditions.

### PhoY1 and PhoY2 are required for stationary-phase survival of *Mycobacterium tuberculosis*.

**FIG 2:**
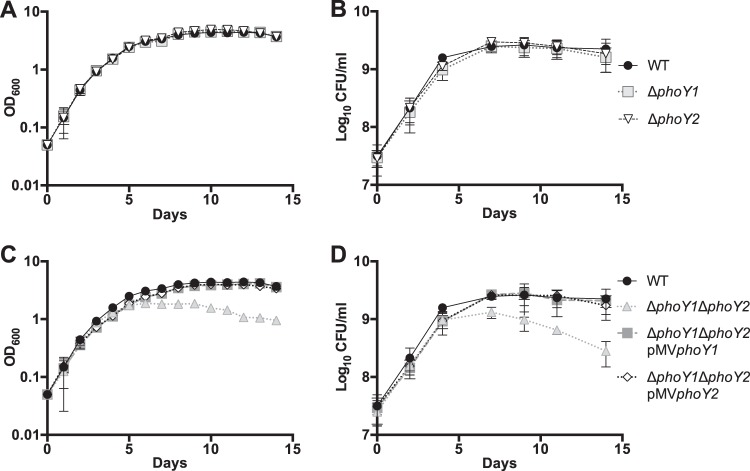
Deletion of *phoY1* and *phoY2* causes a stationary-phase growth defect. *M. tuberculosis* WT and Δ*phoY1*, Δ*phoY2*, Δ*phoY1* Δ*phoY2*, Δ*phoY1* Δ*phoY2/*pMV*phoY1*, and Δ*phoY1* Δ*phoY2*/pMV*phoY2* mutants were grown to mid-exponential phase (OD_600_ of 0.5) in 7H9 medium, diluted to an OD_600_ of 0.05 in fresh 7H9 medium, and incubated at 37°C with shaking. (A and C) Optical density (OD_600_) was measured daily. (B and D) Viable CFU were enumerated by plating serially diluted cultures on 7H10 agar. For all panels, results shown are the average values ± standard deviations of three independent experiments.

Two independently constructed *E. coli* Δ*phoU* mutants exhibited growth defects both on P_i_-rich agar plates and in P_i_-rich liquid medium (9, 11). Specifically, an *E. coli* Δ*phoU* mutant failed to achieve the same overall growth yield in stationary phase, though it grew at the same rate as WT *E. coli* in exponential phase (9). To determine if deletion of *phoY1* or *phoY2* affects *M. tuberculosis* replication, we monitored the growth of mutants in standard P_i_-rich 7H9 medium. We observed neither significant differences in the exponential-phase growth rates (Fig. 2A; Table S1) nor any difference in the growth yields (Fig. 2A and B) of the Δ*phoY1* and Δ*phoY2* mutants compared to that of the WT. The Δ*phoY1* Δ*phoY2* mutant also doubled at a rate similar to that of the WT in exponential phase (Table S1). However, the Δ*phoY1* Δ*phoY2* mutant transitioned to stationary-phase growth earlier than the WT and never achieved the same growth yield (Fig. 2C and D). Both the optical density and viability of Δ*phoY1* Δ*phoY2* cultures slowly declined after entry into stationary phase (Fig. 2C and D). The optical densities of Δ*phoY1* Δ*phoY2* cultures were significantly lower than that of the WT control beginning at day 5 (Fig. 2C). Cultures of the Δ*phoY1* Δ*phoY2* mutant also contained significantly fewer viable CFU than WT cultures beginning at day 7 (Fig. 2D). Although complementation of the Δ*phoY1* Δ*phoY2* mutant with either pMV*phoY1* or pMV*phoY2* caused a modest reduction in the exponential-phase growth rate (Table S1), the complemented strains continued to replicate after 5 days and reached stationary-phase optical densities and viable colony counts similar to those of the WT control (Fig. 2C and D). These data suggest that a functional PhoY1 or PhoY2 protein is necessary for *M. tuberculosis* survival in stationary phase.

10.1128/mBio.00494-17.8 TABLE S1  Doubling times of *phoY* deletion mutants in P_i_-rich 7H9 medium. Download TABLE S1, PDF file, 0.1 MB. Copyright © 2017 Namugenyi et al. 2017 Namugenyi et al. This content is distributed under the terms of the Creative Commons Attribution 4.0 International license.

### Deletion of *phoY1* and *phoY2* increases the sensitivity of *M. tuberculosis* to cell wall and reactive oxygen stress.

A Δ*pstA1* mutant exhibited increased sensitivity to cell wall and oxidative stress *in vitro* due to constitutive activation of RegX3 (16). To determine whether PhoY1 and/or PhoY2 is similarly involved in *M. tuberculosis* resistance to *in vitro* stress conditions, we tested the sensitivity of the *phoY* mutants to the cell wall-disrupting detergent sodium dodecyl sulfate (SDS) and the reactive oxygen species hydrogen peroxide (H_2_O_2_). Deletion of either *phoY1* or *phoY2* alone had no significant effect on the sensitivity of *M. tuberculosis* to SDS or H_2_O_2_ (Fig. S2). In contrast, the Δ*phoY1* Δ*phoY2* mutant was significantly more susceptible than the WT to both SDS and H_2_O_2_, and these phenotypes were reversed by complementation with either *phoY1* or *phoY2* (Fig. S2). These results indicate that PhoY1 or PhoY2 is required for resistance to the SDS and H_2_O_2_ *in vitro* stress conditions.

10.1128/mBio.00494-17.2 FIG S2  The Δ*phoY1* Δ*phoY2* mutant is hypersensitive to cell wall and reactive oxygen stress. The indicated *M. tuberculosis* strains were grown to mid-exponential phase (OD_600_ 0.5) in 7H9 medium, diluted to an OD_600_ of 0.05 in fresh 7H9 medium, and subjected to either sodium dodecyl sulfate (SDS; 0.125%) or hydrogen peroxide (H_2_O_2_; 3 mM) treatment for 24 h at 37°C with shaking. At 0 and 24 h, cultures were serially diluted and plated on 7H10 agar for CFU enumeration. Percent survival was calculated as follows: (CFU poststress)/(CFU prestress) × 100. Results presented are the mean values ± standard errors from three independent experiments. Asterisks indicate statistically significant differences in survival compared to the results for the WT, as follows: **, *P* < 0.005; ***, *P* < 0.0005. Download FIG S2, EPS file, 0.1 MB. Copyright © 2017 Namugenyi et al. 2017 Namugenyi et al. This content is distributed under the terms of the Creative Commons Attribution 4.0 International license.

### Δ*phoY1* Δ*phoY2* bacteria have a lower persister frequency than WT bacteria that is RegX3 dependent.

**FIG 3:**
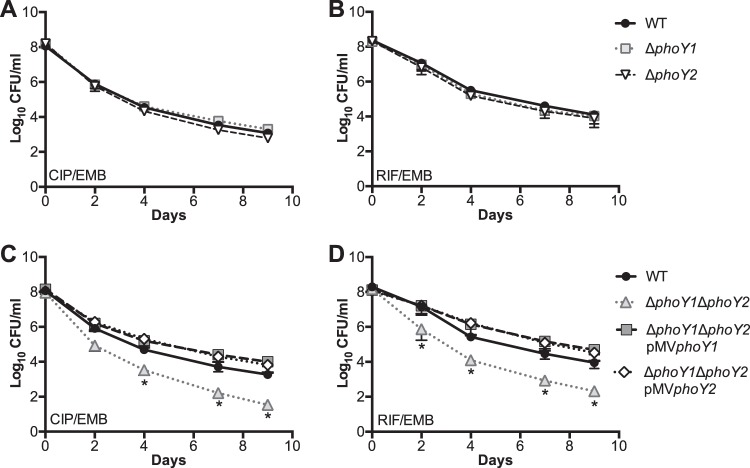
Deletion of *phoY1* and *phoY2* decreases persister frequency in *M. tuberculosis*. The indicated *M. tuberculosis* strains were grown in 7H9 medium to mid-exponential phase (OD_600_ 0.5) and diluted to an OD_600_ of 0.2 before adding antibiotics. Cultures were incubated at 37°C with aeration, and viable CFU/ml were enumerated at the indicated times by plating serial dilutions of cultures on 7H10 agar. Results shown are the average values ± standard deviations of three independent experiments. Asterisks indicate statistically significant differences compared to the results for the WT, as follows: *, *P* < 0.05. (A and C) Ciprofloxacin (CIP) at 8 μg/ml and ethambutol (EMB) at 4 μg/ml. (B and D) Rifampin (RIF) at 0.1 μg/ml and EMB at 4 μg/ml.

**FIG 4:**
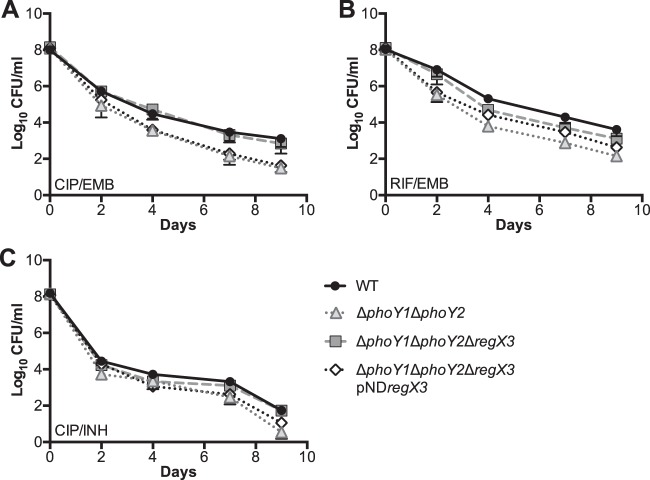
Deletion of *regX3* suppresses the persister defect of the Δ*phoY1* Δ*phoY2* mutant. The indicated *M. tuberculosis* strains were grown in 7H9 medium to mid-exponential phase (OD_600_ 0.5) and diluted to an OD_600_ of 0.2 prior to adding antibiotics. Cultures were incubated at 37°C with shaking, and viable CFU/ml were enumerated at the indicated times by plating serially diluted cultures on 7H10 agar. Results shown are the average values ± standard deviations of three or five independent experiments. (A) Ciprofloxacin (CIP) at 8 μg/ml and ethambutol (EMB) at 4 μg/ml. (B) Rifampin (RIF) at 0.1 μg/ml and EMB at 4 μg/ml. (C) CIP at 8 μg/ml and isoniazid (INH) at 0.1 μg/ml.

*E. coli phoU* was identified as a gene involved in persister formation (8). To determine if *M. tuberculosis phoY1* and/or *phoY2* is similarly required for persister formation, we monitored the survival of bacteria treated with several different antibiotic combinations. The antibiotic combinations consisted of two drugs with different modes of action (rifampin [RIF] and ethambutol [EMB], ciprofloxacin [CIP] and EMB, or CIP and isoniazid [INH]), to prevent the outgrowth of genetically resistant clones. Each combination included a bacteriostatic drug (EMB or low-dose INH) and a bactericidal drug (RIF or CIP) to facilitate persister isolation, as described previously (20). Antibiotic-treated cultures of *M. tuberculosis* typically exhibit biphasic kill kinetics, with initial rapid killing of the nonpersisters followed by a lower death rate, indicative of persister variants present in the initial population (20, 21). We observed characteristic biphasic killing of WT *M. tuberculosis* upon exposure to the antibiotic combinations CIP-EMB and RIF-EMB; the nonpersister population was killed rapidly during the first 4 days, after which the persister subpopulation was killed more slowly (Fig. 3A and B). The Δ*phoY1* and Δ*phoY2* mutants were killed with biphasic kinetics identical to that of the WT during treatment with the CIP-EMB or RIF-EMB antibiotic combination (Fig. 3A and B). In contrast, significantly fewer Δ*phoY1* Δ*phoY2* bacteria than WT bacteria survived treatment with CIP-EMB and RIF-EMB (Fig. 3C and D). The higher rate of death of Δ*phoY1* Δ*phoY2* bacteria over the first 4 days of antibiotic exposure indicates a reduced percentage of the initial population in the persister state. By day 9, there were 54-fold and 43-fold fewer Δ*phoY1* Δ*phoY2* bacteria than WT bacteria in CIP-EMB- and RIF-EMB-treated cultures, respectively. The Δ*phoY1* Δ*phoY2* mutant displayed a trend toward decreased survival compared to that of the WT during treatment with CIP-INH, though the difference was not statistically significant (Fig. 4C). Complementation with either *phoY1* or *phoY2* reversed the persister defect of the Δ*phoY1* Δ*phoY2* mutant (Fig. 3C and D). The Δ*phoY1* Δ*phoY2/*pMV*phoY1* and Δ*phoY1* Δ*phoY2/*pMV*phoY2* complemented strains both survived RIF-EMB and CIP-EMB treatment better than the WT, though the differences were not statistically significant (Fig. 3C and D). These results suggest that both *phoY1* and *phoY2* are required for persister formation in *M. tuberculosis*.

To determine if the decreased persister frequency in Δ*phoY1* Δ*phoY2* bacteria is dependent on RegX3, we analyzed the kill kinetics of a Δ*phoY1* Δ*phoY2* Δ*regX3* mutant using the same three antibiotic combinations (CIP-EMB, RIF-EMB, and CIP-INH). Deletion of *regX3* in the Δ*phoY1* Δ*phoY2* mutant restored the persister frequency in both CIP-EMB- and CIP-INH-treated cultures to WT levels (Fig. 4A and C). Complementation of the Δ*phoY1* Δ*phoY2* Δ*regX3* mutant with pND*regX3* decreased the persister frequency to the same level seen in the Δ*phoY1* Δ*phoY2* mutant in both CIP-EMB- and CIP-INH-treated cultures (Fig. 4A and C). However, in RIF-EMB-treated cultures, the Δ*phoY1* Δ*phoY2* Δ*regX3* mutant exhibited an intermediate phenotype between those of the *phoY* double mutant and the WT, and complementation with pND*regX3* did not fully restore the persister defect characteristic of the *ΔphoY1* Δ*phoY2* mutant (Fig. 4B). Taken together, these data indicate that Δ*phoY1* Δ*phoY2* bacteria have a lower persister frequency that is primarily due to constitutive activation of RegX3 but that other, RegX3-independent mechanisms may contribute to defective persister formation in the Δ*phoY1* Δ*phoY2* mutant under some conditions.

### PhoY1 and PhoY2 are required for persister formation in stationary phase.

Previously, an *M. tuberculosis* H37Rv Δ*phoY2* mutant was reported to have a persister defect in stationary-phase cultures treated with pyrazinamide or RIF (18), suggesting that only PhoY2 and not PhoY1 is involved in persister formation. To test whether PhoY2 is specifically required for persister formation in stationary phase, we monitored the survival of stationary-phase bacteria exposed to a high concentration of RIF (8 μg/ml). At days 3 and 9, there were no significant differences in the survival of Δ*phoY1*, Δ*phoY2*, or Δ*phoY1* Δ*phoY2* Δ*regX3* bacteria compared to that of the WT control (Fig. S3). In contrast, the survival of Δ*phoY1* Δ*phoY2* and Δ*phoY1* Δ*phoY2* Δ*regX3*/pND*regX3* bacteria was reduced compared to that of the WT, with significant differences at day 9 (Fig. S3). These data suggest that PhoY1 and PhoY2 function redundantly to promote *M. tuberculosis* persister formation in stationary phase by inhibiting the activation of RegX3.

10.1128/mBio.00494-17.3 FIG S3  Deletion of *phoY1* and *phoY2* decreases stationary-phase persister frequency. The indicated *M. tuberculosis* strains were diluted to an OD_600_ of 0.05 and grown for 10 days in 7H9 medium to stationary phase before adding 8 μg/ml rifampin. Cultures were incubated at 37°C without shaking. CFU were enumerated at 0, 3, and 9 days by plating serially diluted cultures on 7H10 agar. Percent survival was calculated as follows: (CFU poststress)/(CFU prestress) × 100. Results presented are the mean values ± standard errors from three independent experiments. Asterisks indicate statistically significant differences in survival compared to the results for the WT control, as follows: *, *P* < 0.05. Download FIG S3, EPS file, 0.1 MB. Copyright © 2017 Namugenyi et al. 2017 Namugenyi et al. This content is distributed under the terms of the Creative Commons Attribution 4.0 International license.

### The Δ*phoY1* Δ*phoY2* mutant is hypersusceptible to rifampin but not to other antimycobacterial compounds.

To validate that the decreased persister phenotype we observed in Δ*phoY1* Δ*phoY2* bacteria was not due to reduced intrinsic resistance to antibiotics, we determined the MICs (MIC_90_) of the *phoY* mutants for CIP, EMB, INH, and RIF. The MIC_90_s of the Δ*phoY1* and Δ*phoY2* single mutants were similar to those of the WT for all four drugs (Table 1). In contrast, though Δ*phoY1* Δ*phoY2* bacteria were equally as susceptible as WT bacteria to CIP, EMB, and INH, the RIF MIC_90_ was 4-fold lower than that of the WT (Table 1). Complementation of the Δ*phoY1* Δ*phoY2* mutant with either *phoY1* or *phoY2* in *trans* restored the RIF MIC_90_ to that observed for the WT (Table 1). Deletion of *regX3* in the Δ*phoY1* Δ*phoY2* mutant also partially reversed the RIF sensitivity phenotype (Table 1). Complementation of the Δ*phoY1* Δ*phoY2* Δ*regX3* mutant with pND*regX3* restored the 4-fold-lower RIF MIC_90_ characteristic of Δ*phoY1* Δ*phoY2* bacteria (Table 1). These data suggest that the Δ*phoY1* Δ*phoY2* strain is more susceptible than the WT to RIF due to constitutive activation of RegX3. However, changes in intrinsic resistance cannot explain the decreased tolerance to the CIP-EMB or CIP-INH drug combinations that we observed in the Δ*phoY1* Δ*phoY2* mutant.

**TABLE 1:** MICs of antibiotics against *M. tuberculosis* wild-type and phosphate regulation mutants

Genotype	MIC_90_ (μg/ml) of^a^:			
	CIP	EMB	INH	RIF
WT	0.2	0.5–1	0.05	0.050
Δ*phoY1*	0.2	1	0.025	0.050
Δ*phoY2*	0.2	0.5	0.025	0.050
Δ*phoY1* Δ*phoY2*	0.1–0.2	0.5	0.025	0.0125
Δ*phoY1* Δ*phoY2*/pMV*phoY1*	0.2	0.5	0.025	0.050
Δ*phoY1* Δ*phoY2/*pMV*phoY2*	0.2	0.5	0.025	0.050
Δ*phoY1* Δ*phoY2* Δ*regX3*	0.2	1	0.025	0.025
Δ*phoY1* Δ*phoY2* Δ*regX3/*pND*regX3*	0.2	1	0.025	0.0125
Δ*pstA1*	0.1–0.2	0.5	0.025–0.05	0.00625
Δ*regX3*	0.2	0.5–1	0.025–0.05	0.025–0.05
Δ*pstA1* Δ*regX3*	0.2	0.5–1	0.025–0.05	0.025–0.05
Δ*pstA1*/pMV*pstA1*	—	—	—	0.025–0.05
Δ*pstA1* Δ*regX3*/pND*regX3*	—	—	—	0.00625

^a^ MIC_90_ (μg/ml) is the minimum concentration required to inhibit 90% of growth compared to the results for the no-drug control. Results are from at least three independent experiments. Ranges are given for strains that exhibited variable MIC_90_s in two of four experiments. CIP, ciprofloxacin; EMB, ethambutol; INH, isoniazid; RIF, rifampin; —, MIC_90_ not determined.

Since RIF enters *M. tuberculosis* by diffusion through the cell wall (22), the Δ*phoY1* Δ*phoY2* mutant may exhibit increased susceptibility to this drug due to increased cell envelope permeability. To test this possibility, we performed ethidium bromide uptake assays. We observed a statistically significant 3-fold increase in the ethidium bromide uptake rate for the Δ*phoY1* Δ*phoY2* mutant (15.31 ± 5.09 relative fluorescence units [RFU]/min; *P* = 0.006) relative to that of the WT control (4.66 ± 0.99 RFU/min) (Fig. S4). However, this phenotype was complemented only by *phoY2* (Fig. S4). Since the RIF sensitivity phenotype of the Δ*phoY1* Δ*phoY2* mutant can be complemented either by *phoY1* or *phoY2* (Table 1), these data suggest that a mechanism other than a change in cell envelope permeability is responsible for its RIF sensitivity.

10.1128/mBio.00494-17.4 FIG S4  Increased envelope permeability of the Δ*phoY1* Δ*phoY2* mutant is caused by deletion of *phoY2*. *M. tuberculosis* mc^2^7000, the Δ*phoY1* Δ*phoY2* mutant, and complemented strains were incubated with 2 μg/ml ethidium bromide and uptake rates were determined by measuring emission at 590 nm upon excitation at 544 nm. Data are expressed as relative fluorescence units (RFU) normalized to the fluorescence at 0 min and are the mean values ± standard errors of at least three independent experiments. Download FIG S4, EPS file, 0.1 MB. Copyright © 2017 Namugenyi et al. 2017 Namugenyi et al. This content is distributed under the terms of the Creative Commons Attribution 4.0 International license.

### The Δ*pstA1* mutant exhibits a decrease in persister frequency that is RegX3 dependent.

**FIG 5:**
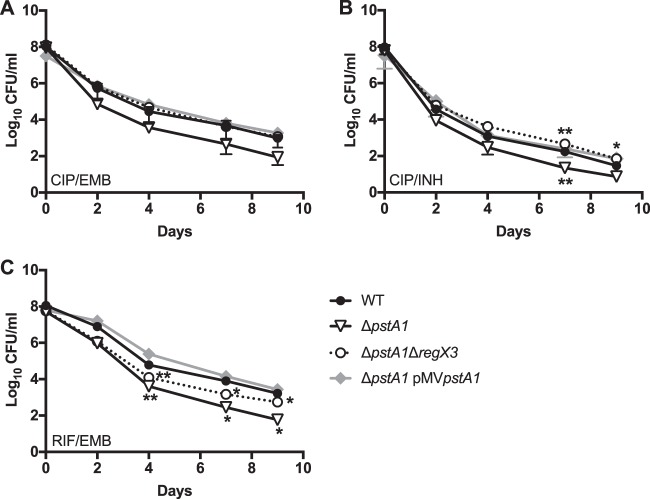
Loss of *pstA1* decreases persister frequency in *M. tuberculosis*. *M. tuberculosis* strains were grown to mid-exponential phase (OD_600_ 0.5), diluted in fresh 7H9 medium to an OD_600_ of 0.2, and then treated with antibiotics. Cultures were incubated with aeration at 37°C, and viable CFU/ml were enumerated at the indicated times by plating serial dilutions on 7H10 agar. Results presented are the average values ± standard errors of three independent experiments. Asterisks indicate statistically significant differences from the results for the WT, as follows: *, *P* < 0.05; **, *P* < 0.005. (A) Ciprofloxacin (CIP) at 8 μg/ml and ethambutol (EMB) at 4 μg/ml. (B) CIP at 8 μg/ml and isoniazid (INH) at 0.1 μg/ml. (C) Rifampin (RIF) at 0.1 μg/ml and at EMB 4 μg/ml.

Since the Δ*phoY1* Δ*phoY2* mutant phenocopies the Δ*pstA1* mutant with respect to gene expression and *in vitro* stress sensitivity, we tested whether the Δ*pstA1* mutant exhibits a similar reduction in persister frequency. We observed a consistent trend of decreased persister frequency for the Δ*pstA1* mutant during treatment with the CIP-EMB, CIP-INH, and RIF-EMB antibiotic combinations (Fig. 5). Complementation with pMV*pstA1* restored WT persister frequency, confirming that these phenotypes were due to the *pstA1* deletion (Fig. 5). Deletion of *regX3* in the Δ*pstA1* background increased the persister frequency to a level comparable to that in the WT (Fig. 5). In fact, the Δ*pstA1* Δ*regX3* mutant survived CIP-INH treatment modestly better than the WT (Fig. 5B). Under the RIF-EMB treatment condition, the persister frequency of the Δ*pstA1* Δ*regX3* mutant, though improved compared to that of the Δ*pstA1* mutant, did not reach the WT level (Fig. 5C), similar to the intermediate phenotype observed for the Δ*phoY1* Δ*phoY2* Δ*regX3* strain (Fig. 4C). Complementation of the Δ*pstA1* Δ*regX3* mutant with pND*regX3* resulted in significantly fewer persisters recovered compared to the level in the WT strain, restoring the Δ*pstA1* mutant phenotype (Fig. S5). These data suggest that constitutive activation of RegX3 in the Δ*pstA1* strain causes decreased persister formation under these nutrient-rich conditions. The deletion of *regX3* did not alter the persister phenotype for either the CIP-EMB or CIP-INH treatment (Fig. S5A and B). However, the Δ*regX3* mutant did have a significant decrease in persister frequency under the RIF-EMB condition that could be complemented (Fig. S5C). These data suggest that RegX3 itself can also influence persister formation.

10.1128/mBio.00494-17.5 FIG S5  Loss of *regX3* decreases *M. tuberculosis* persister frequency when exposed to rifampin and ethambutol. *M. tuberculosis* strains were grown to mid-exponential phase (OD_600_ 0.5) and diluted in fresh 7H9 medium to an OD_600_ of 0.2 prior to the addition of antibiotics. Cultures were incubated with aeration at 37°C, and viable CFU/ml were enumerated at the indicated times by plating serial dilutions on 7H10 agar. Results presented are the average values ± standard errors of three independent experiments. Asterisks indicate statistically significant differences from the results for the WT, as follows: *, *P* < 0.05; **, *P* < 0.005. (A) Ciprofloxacin (CIP) at 8 μg/ml and ethambutol (EMB) at 4 μg/ml. (B) CIP at 8 μg/ml and isoniazid (INH) at 0.1 μg/ml. (C) Rifampin (RIF) at 0.1 μg/ml and EMB at 4 μg/ml. Download FIG S5, EPS file, 0.1 MB. Copyright © 2017 Namugenyi et al. 2017 Namugenyi et al. This content is distributed under the terms of the Creative Commons Attribution 4.0 International license.

It is possible that the Δ*pstA1* mutant exhibits decreased persister frequency simply due to increased sensitivity to the antibiotics. To test this, we determined the MIC_90_s of CIP, RIF, EMB, and INH for each strain. The Δ*pstA1*, Δ*regX3*, and Δ*pstA1* Δ*regX3* strains were either at or within 2-fold of the WT MIC_90_ for all drugs except RIF (Table 1). The Δ*pstA1* strain exhibited an 8-fold increase in sensitivity to RIF, which was complemented by pMV*pstA1*. The Δ*pstA1* Δ*regX3* strain had a nearly wild-type RIF MIC_90_, indicating that RIF sensitivity is RegX3 dependent; the addition of the pND*regX3* vector to this strain restored hypersensitivity to RIF (Table 1). Therefore, the decreased frequency of persisters in the Δ*pstA1* mutant is not simply due to increased sensitivity to CIP, EMB, or INH; however, this cannot be ruled out for RIF.

**FIG 6:**
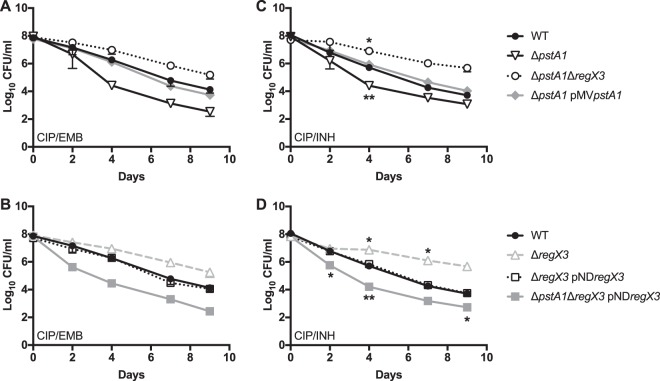
PstA1 is required for *M. tuberculosis* persister formation during phosphate-limiting growth. *M. tuberculosis* strains were grown to mid-exponential phase (OD_600_ 0.5) in complete 7H9 medium, washed twice in P_i_-limiting 7H9 containing 2.5 μM P_i_, and then diluted to an OD_600_ of 0.1 in P_i_-limiting 7H9 containing 2.5 μM P_i_. Cultures were incubated with aeration at 37°C in P_i_-limiting 7H9 for 72 h prior to the addition of antibiotics. Viable CFU/ml were enumerated at the indicated times by plating serial dilutions on 7H10 agar. Results presented are the average values ± standard errors of three independent experiments. Asterisks indicate statistically significant differences from the results for the WT, as follows: *, *P* < 0.05; **, *P* < 0.005. (A and B) Ciprofloxacin (CIP) at 8 μg/ml and ethambutol (EMB) at 4 μg/ml. (C and D) CIP at 8 μg/ml and isoniazid (INH) at 0.1 μg/ml.

The Pst/SenX3-RegX3 system is important for responding to fluctuations in the extracellular P_i_ concentration. Therefore, it seemed plausible that this system might also participate in persister formation during P_i_ limitation. To test this, exponentially growing cultures were subjected to P_i_-limiting conditions for 72 h prior to antibiotic exposure. We chose to provide P_i_ at a concentration (2.5 μM) that would sustain growth but would still activate RegX3-dependent P_i_-responsive genes (23), since the use of P_i_-free medium would result in a slow decline in cell viability (16). During P_i_ limitation, the Δ*pstA1* mutant exhibited a trend of decreased persister frequency for both CIP-EMB and CIP-INH conditions; complementation with pMV*pstA1* restored the WT phenotype (Fig. 6A and C). Unexpectedly, the Δ*pstA1* Δ*regX3* strain had a higher persister frequency than the WT control (Fig. 6A and C). This phenotype was complemented by the pND*regX3* plasmid, resulting in a decreased persister frequency comparable to that of the Δ*pstA1* mutant (Fig. 6B and D). The Δ*regX3* mutant also exhibited a higher persister frequency than the WT, and this phenotype was complemented by the pND*regX3* plasmid (Fig. 6B and D). Because RegX3 is activated during P_i_-limiting conditions, these data indicate that RegX3 activation is detrimental to persister formation. These data further suggest that the Pst system contributes to persister formation in *M. tuberculosis* via an unidentified RegX3-dependent mechanism under both P_i_-rich and P_i_-limiting growth conditions.

### The P_i_-responsive signal transduction system is required for chronic-phase survival and antibiotic tolerance *in vivo*.

**FIG 7:**
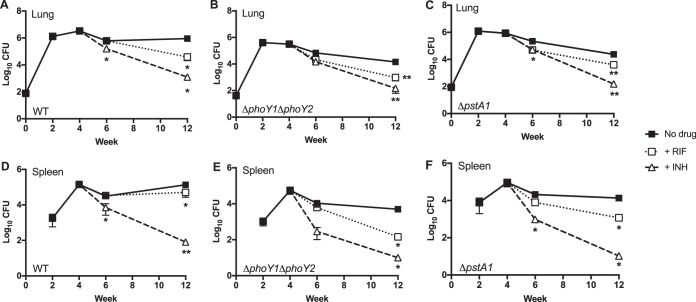
Persistence of Δ*phoY1* Δ*phoY2* and Δ*pstA1* mutants in mice treated with rifampin or isoniazid. C57BL/6 mice were aerosol infected with ~80 CFU of WT Erdman (A and D) and Δ*pstA1* (C and F) strains or ~40 CFU of the Δ*phoY1* Δ*phoY2* mutant (B and E). Four weeks postinfection, mice were divided into no-drug control (closed squares), rifampin treatment (RIF; open squares), and isoniazid treatment (INH; open triangles) groups. At the indicated time points, groups of mice (*n* = 4) were sacrificed, and CFU were enumerated by plating serially diluted lung (A to C) and spleen (D to F) homogenates on 7H10 agar. Results presented are the mean values ± standard errors of the means. Asterisks indicate statistically significant differences compared to the results for the no-drug control, as follows: *, *P* < 0.05; **, *P* < 0.005. For the Δ*pstA1* mutant, the results for both the rifampin and isoniazid treatment groups were significantly different from the results for the no-drug control group in the lungs at week 6 (*P* < 0.05).

Having observed decreased persister frequency in Δ*phoY1* Δ*phoY2* and Δ*pstA1* bacteria in liquid medium, we investigated whether *phoY1*, *phoY2*, or *pstA1* is required for persister formation *in vivo*. C57BL/6 mice were infected with ~100 CFU of WT *M. tuberculosis*, the Δ*phoY1* Δ*phoY2* mutant, or the Δ*pstA1* mutant by the aerosol route. Despite using an ~4-fold-higher dose of the Δ*phoY1* Δ*phoY2* mutant, we obtained on average only 41 CFU per lung at 24 h postinfection (Fig. 7B; Fig. S6A), indicating a possible colonization defect. During the first 2 weeks of infection, each strain grew exponentially in the lungs and disseminated to the spleen (Fig. 7; Fig. S6). The CFU counts of the Δ*phoY1* Δ*phoY2* mutant recovered from the lungs were significantly lower than those of the WT at both the acute (2 and 4 weeks) and chronic phases (6 and 12 weeks) of infection (Fig. 7B; Fig. S6A). The attenuation of the Δ*phoY1* Δ*phoY2* mutant during the chronic phase was comparable to what was observed for the Δ*pstA1* mutant both here (Fig. 7C; Fig. S6A) and previously (16). Despite the growth defect in the lungs, the *ΔphoY1 ΔphoY2* mutant disseminated to the spleen and replicated there with kinetics similar to that of the WT (Fig. 7E; Fig. S6B). At 6 weeks postinfection, the viable CFU counts of Δ*phoY1* Δ*phoY2* bacteria in the spleen began to decrease and were significantly reduced compared to those of the WT control (Fig. 7E; Fig. S6B). The Δ*pstA1* mutant also disseminated to and replicated in the spleen comparably to WT bacteria until 6 weeks postinfection, but significantly fewer CFU of the Δ*pstA1* mutant were recovered from the spleen at 12 weeks (Fig. 7F; Fig. S6B). These results demonstrate that PstA1 and PhoY1 or PhoY2 are required for the survival of *M. tuberculosis* in the lungs and spleen during the chronic phase of infection.

10.1128/mBio.00494-17.6 FIG S6  The Δ*phoY1* Δ*phoY2* mutant is attenuated *in vivo*. C57BL/6 mice were aerosol infected with ~80 CFU of the WT Erdman or Δ*pstA1* strains or ~40 CFU of the Δ*phoY1* Δ*phoY2* mutant. At the indicated time points, groups of mice (*n* = 4) were sacrificed and CFU were enumerated by plating serially diluted lung (A) and spleen (B) homogenates on 7H10 agar. Results shown are the mean values ± standard errors of the means. Asterisks indicate statistically significant differences between the results for the WT and *ΔphoY1 ΔphoY2* or Δ*pstA1* mutants, as follows: *, *P* < 0.05; **, *P* < 0.005. Download FIG S6, EPS file, 0.1 MB. Copyright © 2017 Namugenyi et al. 2017 Namugenyi et al. This content is distributed under the terms of the Creative Commons Attribution 4.0 International license.

Four weeks postinfection, we initiated treatment of groups of mice with either RIF or INH. In mice infected with WT *M. tuberculosis*, RIF caused significant reductions in the bacterial burdens in lungs and spleens only after 8 weeks of treatment (12 weeks postinfection), with comparatively less bacterial clearance in the spleens (Fig. 7A and D). INH treatment of mice infected with WT bacteria caused statistically significant decreases in bacterial loads in both the lungs and spleens after 2 weeks of treatment (6 weeks postinfection) and continued clearance in both tissues through 12 weeks postinfection (Fig. 7A and D). Treatment of mice infected with Δ*phoY1* Δ*phoY2* bacteria with either RIF or INH also resulted in significant reductions in viable CFU counts recovered from both lungs and spleens at 12 weeks postinfection (Fig. 7B and E). Although there was apparently rapid clearance of Δ*phoY1* Δ*phoY2* bacteria from the spleens of INH-treated mice, the decrease in CFU did not quite achieve statistical significance (*P* = 0.0767) (Fig. 7E). Nevertheless, because Δ*phoY1* Δ*phoY2* bacteria exhibited a persistence defect in the absence of a drug, the CFU recovered from the drug-treated mice at 12 weeks postinfection were at least 1 log lower than for the corresponding WT control (Fig. 7). In mice infected with the Δ*pstA1* mutant, RIF treatment caused significant reductions in bacterial burdens in the lungs at both 6 and 12 weeks postinfection (Fig. 7C) and in the spleens at 12 weeks postinfection (Fig. 7F). The bacterial loads of the Δ*pstA1* mutant were also significantly reduced in the lungs and spleens of INH-treated mice at both 6 and 12 weeks postinfection compared to those in the untreated mice (Fig. 7C and F).

Since the Δ*phoY1* Δ*phoY2* and Δ*pstA1* mutants have chronic-phase persistence defects, to enable comparisons of antibiotic tolerance between the mutants and the WT control, we calculated the percentage survival in drug-treated mice relative to the corresponding no-drug control. Both the Δ*phoY1* Δ*phoY2* and Δ*pstA1* mutants were more effectively cleared from the spleens of INH-treated mice at 6 weeks postinfection, but this enhanced susceptibility was no longer apparent at the 12-week time point (Table S2). Both mutants were modestly more susceptible to RIF treatment in the lungs and spleens at 6 weeks postinfection, since there was no reduction in the CFU counts of WT bacteria by RIF at this time point (Table S2). By 12 weeks postinfection, all strains were killed by RIF to a similar extent in the lungs, but in the spleen, both the Δ*phoY1* Δ*phoY2* and Δ*pstA1* mutants were cleared more effectively by RIF than was the WT control (Table S2). These data suggest that the Δ*phoY1* Δ*phoY2* and Δ*pstA1* mutants are moderately more susceptible to RIF during growth in host tissue, particularly in the spleen.

10.1128/mBio.00494-17.9 TABLE S2  Percent survival of bacteria in lungs and spleens of antibiotic-treated mice relative to survival in no-drug control mice. Download TABLE S2, PDF file, 0.1 MB. Copyright © 2017 Namugenyi et al. 2017 Namugenyi et al. This content is distributed under the terms of the Creative Commons Attribution 4.0 International license.

### The Pst/SenX3-RegX3 system controls polyphosphate accumulation during mid-logarithmic growth in *M. tuberculosis*.

Polyphosphate (polyP), a polymer of P_i_ residues linked by high-energy phosphoanhydride bonds, accumulates during starvation for nutrients, including P_i_ (24, 25). In *M. tuberculosis*, polyP accumulation is associated with antibiotic tolerance and persister formation (26, 27), while polyP depletion is associated with reduced antibiotic tolerance (28). The SenX3-RegX3 system may control polyP accumulation, since the expression of *ppk1*, which encodes the *M. tuberculosis* polyP kinase, increases during P_i_ limitation and RegX3 binds the *ppk1* promoter (29). Thus, differences in polyP storage may account for the changes in persister formation that we observed in the Pst/SenX-RegX3 system mutants. Therefore, we quantified polyP in the Pst/SenX-RegX3 system mutants during mid-logarithmic growth, a condition in which *M. tuberculosis* does not normally accumulate polyP (28). Both the Δ*phoY1* Δ*phoY2* and Δ*pstA1* mutants stored significantly more polyP than WT bacteria (Table 2). The Δ*phoY1* Δ*phoY2* mutant consistently stored more polyP than the Δ*pstA1* mutant, though the difference was not statistically significant (*P* = 0.054). Complementing the Δ*phoY1* Δ*phoY2* mutant with either *phoY1* or *phoY2* restored a WT level of polyP; the pMV*pstA1* vector similarly complemented the Δ*pstA1* mutant phenotype (Table 2). The accumulation of polyP in the Δ*pstA1* mutant was also RegX3 dependent. The Δ*pstA1* Δ*regX3* mutant stored an amount of polyP similar to that stored by the WT control; complementation with pND*regX3* restored a high level of polyP storage characteristic of the Δ*pstA1* mutant (Table 2). Deletion of *regX3* alone had no impact on polyP storage under the P_i_-rich growth condition we used (Table 2). These data demonstrate that the Pst system inhibits polyP accumulation during nutrient-rich conditions in a RegX3-dependent manner.

**TABLE 2:** Polyphosphate quantification in *M. tuberculosis* wild-type and Pst/SenX3-RegX3 mutant strains^a^

Strain	nmol polyP/mg total protein (mean ± SD)^b^	*P* value (versus WT)
WT	0.09 ± 0.02	
Δ*phoY1*	0.13 ± 0.06	0.258
Δ*phoY2*	0.41 ± 0.17	0.010
Δ*phoY2/*pMV*phoY2*	0.19 ± 0.06	0.023
Δ*phoY1* Δ*phoY2*	0.86 ± 0.21	0.0003
Δ*phoY1* Δ*phoY2*/pMV*phoY1*	0.08 ± 0.02	0.413
Δ*phoY1* Δ*phoY2/*pMV*phoY2*	0.07 ± 0.02	0.205
Δ*phoY1* Δ*phoY2* Δ*regX3*	0.64 ± 0.18	0.001
Δ*phoY1* Δ*phoY2* Δ*regX3*/pND*regX3*	0.44 ± 0.10	0.0003
Δ*pstA1*	0.56 ± 0.15	0.001
Δ*pstA1*/pMV*pstA1*	0.21 ± 0.10	0.051
Δ*regX3*	0.07 ± 0.02	0.306
Δ*pstA1* Δ*regX3*	0.09 ± 0.05	0.957
Δ*pstA1* Δ*regX3*/pND*regX3*	0.45 ± 0.22	0.016

^a^ Strains were grown in 20 ml of 7H9 broth until mid-exponential growth phase (OD_600_ of 0.5) and pelleted by centrifugation prior to polyphosphate extraction.

^b^ Results are mean values ± standard deviations of at least four independent experiments.

In contrast to the Δ*pstA1* mutant, polyP accumulation in the Δ*phoY1* Δ*phoY2* mutant was largely RegX3 independent. Deletion of *regX3* in the Δ*phoY1* Δ*phoY2* strain caused a modest but statistically insignificant decrease in the polyP concentration (*P* = 0.156). Δ*phoY1* Δ*phoY2* Δ*regX3* mutant bacteria still had a significantly elevated polyP concentration relative to the level in the WT control (Table 2). Interestingly, the Δ*phoY2* mutant also stored significantly more polyP than either WT bacteria (Table 2) or the Δ*phoY1* mutant (*P* = 0.022), but this phenotype was only partially complemented by pMV*phoY2* (*P* = 0.058). These data suggest a RegX3-independent role of the PhoY proteins, particularly PhoY2, in controlling polyP production and/or storage.

To determine if changes in polyP storage in the *phoY* mutants were due to altered transcription of genes encoding enzymes involved in polyP synthesis (*ppk1* and *ppk2*) or hydrolysis (*ppx1* or *ppx2*), we performed qRT-PCR. Only the transcription of *ppk1* was significantly changed (Fig. S7). *ppk1* expression was increased 3-fold in the Δ*phoY1* Δ*phoY2* mutant, and this phenotype could be complemented by either *phoY1* or *phoY2* (Fig. S7). RegX3 positively regulates *ppk1* transcription (29). The transcription of *ppk1* was reversed to the WT level in the Δ*phoY1* Δ*phoY2* Δ*regX3* mutant; complementation of the *regX3* deletion restored the 3-fold-higher *ppk1* transcript level characteristic of the Δ*phoY1* Δ*phoY2* mutant (Fig. S7). These data suggest that increased polyP storage by the Δ*phoY1* Δ*phoY2* mutant is partially due to increased synthesis by PPK1 but that posttranscriptional regulation of polyP synthesis or hydrolysis contributes to the increased polyP storage observed in the Δ*phoY2* and Δ*phoY1* Δ*phoY2* Δ*regX3* mutants.

10.1128/mBio.00494-17.7 FIG S7  *ppk1* is overexpressed in the Δ*phoY1* Δ*phoY2* mutant. RNA was isolated from the indicated *M. tuberculosis* strains grown to mid-exponential phase (OD_600_ 0.5) in 7H9 medium. Expression of *ppk1*, *ppk2*, *ppx1*, and *ppx2* transcripts was measured using quantitative reverse transcription-PCR and normalized to the *sigA* transcript level. Data shown are the mean values ± standard deviations of three independent experiments. Asterisks indicate statistically significant differences in transcript abundance compared to the results for the WT, as follows: *, *P* < 0.05. Download FIG S7, EPS file, 0.2 MB. Copyright © 2017 Namugenyi et al. 2017 Namugenyi et al. This content is distributed under the terms of the Creative Commons Attribution 4.0 International license.

## DISCUSSION

Persisters have been implicated in the long-term treatment required to cure *M. tuberculosis* infections, but the mechanisms underlying their formation and survival are not fully understood. Here, we demonstrate that *M. tuberculosis* PhoY1 and PhoY2 play a redundant role in persister formation. Both PhoY proteins function to prevent activation of the P_i_-sensing SenX3-RegX3 two-component system when P_i_ is readily available. This P_i_-signaling function of the PhoY proteins is critical for promoting persister formation, since both the gene expression and persister defects of the Δ*phoY1* Δ*phoY2* mutant could be reversed by deletion of *regX3*. The reduced frequency of persister variants in Δ*phoY1* Δ*phoY2* mutant cultures is not due to decreased intrinsic resistance to antibiotics, since MIC assays indicated little change in susceptibility to the drugs we tested, with the exception of RIF. Furthermore, we observe an increase in persister frequency in cultures of the complemented strains that overexpress either *phoY1* or *phoY2*, a phenomenon previously associated with other *M. tuberculosis* persister genes (30). Our data therefore suggest that *phoY1* and *phoY2* are *bona fide M. tuberculosis* persister genes. Our data further suggest that PhoY1 and PhoY2 mediate persister formation by controlling the activation of RegX3.

Our results contrast with a previous study that suggested only PhoY2 is involved in persister formation (18). Our Δ*phoY2* mutant had no persister defect under any antibiotic treatment condition we tested, including a condition identical to that reported previously. This discrepancy could be due to differences in the *M. tuberculosis* strains used. Alternatively, it is possible that a secondary mutation in the H37Rv Δ*phoY2* strain was responsible for the persister defects, since complementation analysis was not done in the previous study (18). 

We previously demonstrated that the deletion of *pstA1*, which encodes a Pst system transmembrane component, causes constitutive activation of RegX3 (16). Here we show that, like the Δ*phoY1* Δ*phoY2* mutant, Δ*pstA1* bacteria are more susceptible to several different drug combinations *in vitro* and this phenotype is dependent on RegX3. Thus, inhibiting RegX3 activation when P_i_ is abundant is necessary for *M. tuberculosis* persister formation. Our data also suggest that RegX3 itself controls persister formation. A Δ*regX3* mutant exhibits increased persister frequency during growth under P_i_-limiting conditions, a condition in which RegX3 is normally activated to regulate the transcription of P_i_-responsive genes (14, 16, 23). This suggests that whether RegX3 is activated by low P_i_ or by disrupted signaling between the Pst system and SenX3-RegX3, it functions to inhibit the formation of persisters. Further work is required to identify the RegX3-regulated gene or genes that directly influence persister formation.

We used a mouse infection model to determine if disrupting Pst/SenX3-RegX3 signaling causes a similar decrease in antibiotic tolerance *in vivo*. Our results demonstrate that PhoY1 and PhoY2 are required for replication and chronic-phase survival of *M. tuberculosis* in the lungs and spleens of aerosol-infected mice. Additionally, the Δ*phoY1* Δ*phoY2* mutant may be more susceptible to either innate immune responses or the aerosolization procedure, since we were not able to achieve an equivalent input dose, despite multiple attempts. We observed a modest improvement in the clearance of both the Δ*pstA1* and the Δ*phoY1* Δ*phoY2* mutant in mice treated with RIF, particularly in the spleen. This is consistent with the enhanced susceptibility to RIF that we observed by MIC testing *in vitro*. It is possible that the concentration of RIF achieved in the spleen is sufficient to kill the Δ*pstA1* and Δ*phoY1* Δ*phoY2* mutants but not WT bacteria. Others have similarly observed reduced efficacy of RIF against *M. tuberculosis* in the spleen compared to the lungs (31). It is unknown whether this difference in RIF efficacy reflects differences in RIF penetration into lung versus spleen tissue. The antibiotic sensitivity phenotypes that we observed for the Δ*phoY1* Δ*phoY2* and Δ*pstA1* mutants were less pronounced *in vivo*, suggesting that the host immune response may eliminate the same subset of bacteria that are also more susceptible to antibiotics. Nevertheless, there is improved clearance of Δ*phoY1* Δ*phoY2* and Δ*pstA1* mutant bacteria from infected tissues due to the combined effect of the host immune response and RIF treatment.

Since RIF enters *M. tuberculosis* by diffusion through the mycobacterial cell wall (22), we hypothesized that hypersusceptibility of the Δ*phoY1* Δ*phoY2* and Δ*pstA1* mutants to this drug might be due to increased cell wall permeability. Indeed, both mutants are also hypersusceptible to detergent and reactive oxygen stress, phenotypes that have previously been associated with decreased cell wall integrity (16, 32). Using ethidium bromide uptake assays, we demonstrated that the Δ*phoY1* Δ*phoY2* mutant exhibits enhanced cell wall permeability. We previously made similar observations for the Δ*pstA1* mutant (33). However, there are two lines of evidence that suggest increased envelope permeability is not responsible for the RIF hypersensitivity of these mutants. First, though we could complement the RIF susceptibility of the Δ*phoY1* Δ*phoY2* mutant with *phoY1* or *phoY2*, the cell wall permeability phenotype was complemented only by *phoY2*. Second, we previously demonstrated that the increased envelope permeability of the Δ*pstA1* mutant was attributable to RegX3-dependent overexpression of *pe19*, which encodes a member of the mycobacterial PE protein family (33), but the Δ*pstA1* Δ*pe19* mutant has a RIF MIC_90_ similar to that of the Δ*pstA1* mutant (unpublished data). Together, these data suggest that other RegX3-regulated factor(s) contribute to the RIF sensitivity of the Δ*pstA1* and Δ*phoY1* Δ*phoY2* mutants. Future studies will focus on identifying these RegX3-regulated factor(s).

Drug tolerance and increased persister frequency are often associated with reduced growth rates, such as that observed during stationary phase (21, 34, 35). It is therefore surprising that persister frequency was reduced in both exponential and stationary-phase cultures of the Δ*phoY1* Δ*phoY2* mutant despite the fact that this mutant has a growth defect that causes early entry into stationary phase. These observations suggest that mechanisms other than a change in growth rate may contribute to the persister defect in the Δ*phoY1* Δ*phoY2* mutant. PolyP accumulation has also previously been associated with increased persister frequency in both *E. coli* and *M. tuberculosis* (26–28, 36, 37). In *E. coli*, polyP activates the Lon protease that degrades antitoxins of toxin-antitoxin systems, freeing the toxins to inhibit growth (36, 37). We show that the Δ*phoY1* Δ*phoY2* and Δ*pstA1* mutants both accumulate polyP, yet these mutants also exhibit decreased persister frequency. Furthermore, although deletion of *regX3* in the Δ*phoY1* Δ*phoY2* mutant restored the persister frequency to wild-type levels under most drug treatment conditions, it did not fully suppress the accumulation of polyP. Our data therefore suggest that *M. tuberculosis* has additional mechanisms besides reduced growth rate and polyP accumulation that promote persister formation. Further study will be required to precisely define these molecular mechanisms.

Our data suggest that the *M. tuberculosis* PhoY proteins function redundantly to regulate the activity of SenX3-RegX3 and promote persister formation. Based on the *E. coli* model, both PhoY1 and PhoY2 may be able interact directly with the Pst system and SenX3 to facilitate communication between these systems. Our future studies will explore this possibility. It is also possible that the two *M. tuberculosis* PhoY proteins have evolved additional unique functions or operate under different growth conditions. Indeed, we observed that the Δ*phoY2* single mutant accumulated significantly more polyP than either WT *M. tuberculosis* or the Δ*phoY1* mutant during exponential growth. Similar polyP accumulation was previously observed for a *Mycobacterium marinum phoY2*::Tn mutant (19), suggesting that this function of PhoY2 in regulating polyP synthesis or storage is conserved. Our data also suggest a unique function for PhoY2 in regulating envelope permeability. In other organisms, mutation of *phoU* leads to the accumulation of polyP due to increased uptake of P_i_ from the medium (38, 39). In *E. coli*, PhoU is not required for P_i_ transport but may regulate the P_i_ transport activity of the Pst system (9, 11). The *M. tuberculosis* PhoY proteins may similarly regulate P_i_ transport to influence the accumulation of polyP. *M. tuberculosis* is unusual but not unique in encoding two PhoU orthologs. In *Streptococcus pneumoniae*, which also has two PhoU proteins and two Pst transporters, the PhoU proteins have distinct functions: PhoU2 inhibits P_i_ transport by the Pst2 transporter and controls the activity of the two-component system PnpRS, while PhoU1 only regulates P_i_ transport by the Pst1 transporter (40). While our data indicate that both PhoY1 and PhoY2 function redundantly to promote persister formation by controlling the activity of SenX3-RegX3, it is possible that these proteins have differing abilities to interact with the two *M. tuberculosis* Pst systems to control P_i_ uptake. Our future studies will include characterizing the molecular functions of the PhoY proteins to determine whether they directly influence P_i_ uptake or other functions related to polyP synthesis or storage.

## MATERIALS AND METHODS

### Bacterial culture conditions.

*M. tuberculosis* strain Erdman and derivative strains were grown at 37°C in Middlebrook 7H9 (Difco) liquid culture medium supplemented with 10% albumin-dextrose-saline (ADS), 0.5% glycerol, and 0.1% Tween 80 (complete 7H9) or on Middlebrook 7H10 (Difco) solid culture medium supplemented with 10% oleic acid-albumin-dextrose-catalase (OADC; BD Biosciences) and 0.5% glycerol. *M. tuberculosis* strain mc^2^7000 (H37Rv ΔRD1 Δ*panCD*) and derivatives were cultured using complete 7H9 or 7H10 medium supplemented with 50 μg/ml pantothenic acid (Sigma). Frozen stocks were prepared by growing cultures to mid-exponential phase (OD_600_ of 0.6 to 0.8), adding glycerol to a 15% final concentration, and storing aliquots at −80°C. For P_i_-limiting 7H9 broth (2.5 μM P_i_ 7H9), a 10× liquid stock of 7H9 base was reconstituted without the addition of the P_i_-buffering components. The 1× P_i_-free 7H9 was made with 0.5% glycerol, 10% ADS, 0.1% Tween 80, and 50 mM MOPS [3-(*N*-morpholino)propanesulfonic acid] buffer, pH 6.6, and 2.5 μM KH_2_PO_4_ was added. Antibiotics were used at the following concentrations, unless otherwise indicated: kanamycin at 15 μg/ml; hygromycin at 50 μg/ml; ciprofloxacin (CIP) at 8 μg/ml; rifampin (RIF) at 0.1 μg/ml; ethambutol (EMB) at 4 μg/ml; and isoniazid (INH) at 0.1 μg/ml.

### Cloning.

Constructs for deletion of *phoY1* (*Rv3301c*) or *phoY2* (*Rv0821c*) in *M. tuberculosis* were generated in the allelic exchange vector pJG1100 (41). Genomic regions 800 to 900 bp upstream and downstream from *phoY1* and *phoY2* were PCR amplified from *M. tuberculosis* Erdman genomic DNA using the oligonucleotides listed in Table S3 in the supplemental material. Reverse primers for amplification of the upstream regions were designed with an SphI restriction site in-frame with the translation start codon; the corresponding forward primers for amplification of the downstream regions were designed with an SphI restriction site in-frame with the stop codon. PCR products were cloned in pCR2.1-TOPO (Invitrogen) and sequenced. The upstream and downstream regions were removed from pCR2.1 by restriction with PacI/SphI and SphI/AscI, respectively, and then ligated together in pJG1100 between the PacI and AscI sites to generate the in-frame deletion constructs pAT208 (Δ*phoY1*) and pAT209 (Δ*phoY2*).

10.1128/mBio.00494-17.10 TABLE S3  Oligonucleotide primers used for cloning, strain construction, or real-time quantitative RT-PCR (qRT-PCR). Download TABLE S3, PDF file, 0.1 MB. Copyright © 2017 Namugenyi et al. 2017 Namugenyi et al. This content is distributed under the terms of the Creative Commons Attribution 4.0 International license.

Vectors for complementation of the *phoY* deletions were constructed in the episomal plasmid pMV261 under the control of the native *phoY* promoter. The *phoY1* and *phoY2* genes, including 188 bp or 158 bp 5′ of the translational start site, respectively, were PCR amplified with the primers indicated in Table S3. PCR products were cloned in pCR2.1-TOPO and sequenced. The cloned genes were removed from pCR2.1 by restriction with XbaI and HindIII and ligated into similarly digested pMV261 to generate pMV*phoY1* and pMV*phoY2*.

### Strain construction.

*M. tuberculosis* Δ*phoY1* and Δ*phoY2* deletion mutants were generated by a two-step homologous recombination method for allelic exchange, essentially as described previously (16). Integration of the vectors was confirmed with the following primer pairs, listed in Table S3: Δ*phoY1* upstream Y1F3/Y1R4, Δ*phoY1* downstream Y1seqF/Y1R3, Δ*phoY2* upstream Y2F3/dPTF2, and Δ*phoY2* downstream PTF4/Y2R4. Identification of deletion mutants was done with the following primer pairs: Δ*phoY1* Y1F3/Y1R3 and Δ*phoY2* Y2F3/PTF4. The double-deletion Δ*phoY1* Δ*phoY2* mutant was generated similarly, by electroporating Δ*phoY1* with the pAT209 Δ*phoY2* allelic exchange vector. Deletions were further confirmed by Southern blotting. The triple Δ*phoY1* Δ*phoY2* Δ*regX3* mutant was constructed by electroporating the Δ*phoY1* Δ*phoY2* mutant with the Δ*regX3* allelic exchange vector and screening for the deletion as described previously (16). Complemented strains were constructed by electroporating the corresponding deletion mutants with the pMV*phoY1*, pMV*phoY2*, or pND*regX3* plasmid (16) and selecting on 7H10 medium containing Kan. The presence of the complementing plasmids was confirmed by PCR using the primers listed in Table S3. To analyze the effects of the *phoY* deletions on cell wall permeability, the Δ*phoY1* Δ*phoY2* mutant and complemented derivatives were similarly constructed in the mc^2^7000 attenuated strain. The Δ*pstA1*, Δ*pstA1/*pMV*pstA1*, Δ*regX3*, Δ*pstA1* Δ*regX3*, Δ*regX3*/pND*regX3*, and Δ*pstA1* Δ*regX3*/pND*regX3* mutant strains were described previously (16).

### Southern hybridization.

Genomic DNA extraction and Southern blotting were performed as described previously (33) using the ECL direct nucleic acid labeling kit (Amersham), except that genomic DNA was digested with either PstI (Δ*phoY1*) or XhoI (Δ*phoY2*) and probes were amplified by PCR from *M. tuberculosis* Erdman genomic DNA using the Y1PF/Y1PF (Δ*phoY1*) or TY2PF/TY2PR (Δ*phoY2*) primers, listed in Table S3. Blots were imaged on an Odyssey Fc imager (LI-COR Biosciences).

### Growth curves.

*M. tuberculosis* Erdman and derivative strains were grown to mid-exponential phase (optical density at 600 nm [OD_600_] of 0.5) and then diluted to an OD_600_ of 0.05 in 10 ml of 7H9 medium. Cultures were incubated with aeration at 37°C. Growth was monitored by daily measurement of the OD_600_ and by enumerating CFU at 0, 2, 4, 7, 9, 11, and 14 days by plating serially diluted culture aliquots on 7H10 agar.

### qRT-PCR.

Bacteria were grown to mid-exponential phase (OD_600_ of 0.5) in 7H9 broth, and RNA was extracted as described previously (16). Equivalent amounts of total RNA were treated with Turbo DNase (Ambion) and reverse transcribed to cDNA with the Transcriptor first-strand cDNA synthesis kit (Roche) as described previously (33). Primers for real-time quantitative reverse transcription (qRT)-PCR (Table S3) were designed using Primer Express software (Applied Biosystems) and were tested in standard PCRs using 100 *M. tuberculosis* genome equivalents as the template. Quantitative real-time PCRs were prepared and run in absolute quantification mode on a LightCycler 480 (Roche) as previously described (33). Crossing-point (*C_p_*) PCR cycle values were converted to copy numbers using standard curves for each gene. Target cDNA was internally normalized to *sigA* cDNA.

### Cell wall and ROS stress.

Bacteria were grown to mid-exponential phase (OD_600_ of 0.5) in 7H9 broth, diluted to an OD_600_ of 0.05 in fresh 7H9 broth, and incubated at 37°C after the addition of 0.125% SDS or 3 mM H_2_O_2_. CFU were enumerated at 0 and 24 h by plating serially diluted culture aliquots on 7H10 agar.

### Persister assay.

Bacteria were grown to mid-exponential phase (OD_600_ of 0.5) in 30 ml of 7H9 medium and diluted to an OD_600_ of 0.2 in 50 ml fresh 7H9 medium. Antibiotics were added, and four 12-ml aliquots of the culture were prepared in 30-ml square bottles (Nalgene). Cultures were incubated at 37°C with aeration. At each time point, viable CFU were enumerated using an independent culture bottle. Bacteria in a 1-ml aliquot of the culture were collected by centrifugation (5,000 × *g*), washed once in phosphate-buffered saline (PBS) containing 0.05% Tween 80 (PBS-T), serially diluted, and plated on 7H10 agar. Colonies were counted after 3 to 4 weeks of incubation at 37°C.

For P_i_-limiting growth conditions, bacteria were grown to mid-exponential phase in 7H9 medium, washed once with 2.5 μM P_i_ 7H9, resuspended in 2.5 μM P_i_ 7H9 to an OD_600_ of 0.1, and incubated at 37°C with aeration for 72 h prior to the addition of antibiotics. Bacteria were pelleted (2,850 × *g* for 10 min) and diluted to an OD_600_ of 0.2 using spent medium. Antibiotics were added, and four 12-ml aliquots of the culture were prepared in 30-ml square bottles (Nalgene). Viable CFU remaining at the indicated time points were determined by plating washed and serially diluted cultures as described above.

Stationary-phase persister assays were done with bacteria grown for 10 days in 7H9 medium. Cultures were diluted to an OD_600_ similar to that of the Δ*phoY1* Δ*phoY2* mutant by removing excess culture, pelleting bacteria by centrifugation, and adding back the spent 7H9 medium. Rifampin (8 μg/ml) was added, and cultures were incubated at 37°C without shaking in a CO_2_ incubator. At each time point, culture aliquots were collected and viable CFU were quantified as described above.

### MIC assay.

Bacteria were grown to mid-exponential phase (OD_600_ of 0.5) in 7H9 broth and diluted to an OD_600_ of 0.01 in 5 ml fresh 7H9. Antibiotics were added to the cultures in 2-fold increasing concentrations; cultures without antibiotics were included as controls. Cultures were incubated at 37°C with aeration for 7 days (INH) or 14 days (RIF, EMB, or CIP), and the OD_600_ of each culture was measured. The MIC_90_ was defined as the minimum concentration of antibiotic required to inhibit growth by at least 90% relative to that of the no-antibiotic control.

### Mouse infections.

Seven-week-old female C57BL/6J mice (Jackson Laboratory) were infected with ~100 CFU of *M. tuberculosis* by the aerosol route using an inhalation exposure system (Glas-Col) as described previously (33). Bacterial suspensions used for infection were prepared from cultures grown to mid-exponential phase (OD_600_ of 0.5) in 7H9 broth by washing bacteria once in PBS-T, removing clumps by low-speed centrifugation (150 × *g* for 5 min), and adjusting the declumped supernatant to an OD_600_ of 0.005 (WT or Δ*pstA1*) or 0.02 (Δ*phoY1* Δ*phoY2*) in PBS-T. After 4 weeks of infection, groups of mice were either left untreated or treated with RIF (10 mg/kg of body weight/day) or INH (25 mg/kg/day) provided fresh in the drinking water every 48 to 72 h. At the indicated time points, groups of mice (*n* = 4) were euthanized by CO_2_ overdose for determination of viable CFU in lungs and spleen. CFU were enumerated by plating serially diluted organ homogenates on 7H10 agar containing 100 μg/ml cycloheximide and counting colonies after 3 to 4 weeks of incubation at 37°C. All animal protocols were reviewed and approved by the University of Minnesota Institutional Animal Care and Use Committee and were done in strict accordance with the NIH Guidelines for the Care and Use of Laboratory Animals (42).

### Polyphosphate extraction and quantification.

Polyphosphate (polyP) was extracted from *M. tuberculosis* as described previously (43) with slight modifications. Bacteria grown to mid-logarithmic phase (OD_600_ of 0.4 to 0.7) in 20 ml of 7H9 were pelleted (4,700 × *g* for 15 min) and stored at −80°C until polyP was extracted. Cells were resuspended in 0.9 ml of PBS (Gibco), transferred to 2-ml screw-cap tubes containing 250 μl of 0.1-mm zirconia-silica beads (BioSpec Products), and disrupted by bead beating for 4 min using a Disruptor Genie (Scientific Industries). Beads were pelleted (600 × *g* for 5 min), supernatants were transferred to 1.5-ml screw-cap tubes, and cell debris was removed by centrifugation (3,000 × *g* for 10 min). Supernatants were passed through a 0.22-μm cellulose acetate micro-spin filter (Thermo Fisher) by centrifugation (14,000 × *g* for 3 min) to remove any remaining bacteria. Then, 0.5 ml of GITC (4M guanidine isothiocyanate, 50 mM Tris-HCl [pH 7.0]) lysis buffer prewarmed to 95°C was added, and extracts were incubated at 95°C for 30 min A 10-μl sample was removed for total protein quantification (Pierce bicinchoninic acid [BCA] protein concentration assay; Thermo Scientific). Subsequently, 30 μl of 10% SDS, 500 μl of 95% ethanol, and 5 μl of Glassmilk (GeneClean) were added to each sample and vortex mixed. The Glassmilk was pelleted by brief centrifugation and then resuspended in 500 μl of ice-cold wash buffer (5 mM Tris-HCl [pH 7.5], 50 mM NaCl, 5 mM EDTA, 50% ethanol) by vortexing. Pelleting and washing were repeated twice. The washed Glassmilk was resuspended in 50 μl of 50 mM Tris-HCl (pH 7.4), 10 mM MgCl_2_ containing 20 μg/ml DNase (Roche) and 20 μg/ml RNase (Roche) and incubated at 37°C for 30 min. The Glassmilk was pelleted, washed once with 150 μl of GITC lysis buffer and 150 μl of 95% ethanol, and then washed twice with 300 μl of wash buffer. PolyP was eluted by resuspending Glassmilk in 50 μl of 50 mM Tris-HCl (pH 8.0) and incubating at 95°C for 2 min. Three elutions were performed on each Glassmilk pellet. To quantify polyP, 10 μl of each elution was added to 90 μl of TBO (6 mg/liter toluidine blue O [Sigma] in 40 mM acetic acid) dye solution and incubated for 15 min at room temperature. The binding of TBO to polyP causes a shift in absorbance from 630 nm to 530 nm. Absorbance at 530 nm and 630 nm was measured using a Synergy H1 Hybrid plate reader (BioTek). The *A*_530_/*A*_630_ ratios were compared to a standard curve generated using sodium phosphate glass type 45 (Sigma) to calculate the polyP concentration. Total polyP was normalized to total protein (mg/ml).

### Ethidium bromide uptake.

Ethidium bromide uptake was measured as previously described (27). *M. tuberculosis* mc^2^7000 and derivative strains were grown to mid-exponential phase (OD_600_ of 0.4 to 0.6), pelleted by centrifugation, washed once with PBS-T, and resuspended in PBS-T to an OD_600_ of 0.4 to 0.5. Ethidium bromide was added at a 2-μg/ml final concentration, and uptake was measured using black, flat-bottom, 96-well microplates (Corning) and a Synergy H1 Hybrid plate reader (BioTek) in top-reading mode with excitation at 544 nm and emission at 590 nm. Uptake rates were determined using data in the linear range between 0 and 30 min and are the mean values ± standard deviations of at least three independent experiments.

### Statistical analysis.

Sample sizes for animal experiments were determined by a power calculation. Assuming a typical standard deviation of 35 to 40% of the sample mean, a sample size of *n* = 4 is sufficient to detect a 10-fold (1 log) difference in CFU between groups with a type I error rate (α) of 0.05% to achieve 90% power (44). Student’s unpaired *t* test (two tailed) was used for pairwise comparisons between WT and mutant strains of *M. tuberculosis*. *P* values were calculated using GraphPad Prism 5.0 software (GraphPad Software, Inc.). *P* values of <0.05 were considered significant.
