# Supplementary material for: Mycobacterium tuberculosis PhoY Proteins Promote Persister Formation by Mediating Pst/SenX3-RegX3 Phosphate Sensing
Source: mBio. 2017 Jul 11;8(4):e00494-17. doi: 10.1128/mBio.00494-17 (PMC5513712; doi:10.1128/mBio.00494-17)
Supplement: TABLE S1 [file mbo003173376st1.pdf]

**Table S1.** Doubling times of *phoY* deletion mutants in $P_i$-rich 7H9 medium[a]

| | **Doubling time[b] (hours)** | ***P* value (vs WT)** |
|---|---|---|
| WT | 19.50 ± 1.09 | |
| Δ*phoY1* | 19.69 ± 0.67 | 0.8104 |
| Δ*phoY2* | 19.75 ± 0.77 | 0.7670 |
| Δ*phoY1*Δ*phoY2* | 20.70 ± 0.56 | 0.1645 |
| Δ*phoY1*Δ*phoY2* pMV*phoY1* | 21.66 ± 0.14 | 0.0271 |
| Δ*phoY1*Δ*phoY2* pMV*phoY2* | 21.50 ± 0.28 | 0.0371 |

[a] Doubling times were determined from optical density measurement taken during the first 96 hours of growth after dilution to an $OD_{600}$ of 0.05 in 7H9 medium.

[b] Means ± standard deviations of doubling times from three independent cultures.
