# Supplementary material for: Mycobacterium tuberculosis PhoY Proteins Promote Persister Formation by Mediating Pst/SenX3-RegX3 Phosphate Sensing
Source: mBio. 2017 Jul 11;8(4):e00494-17. doi: 10.1128/mBio.00494-17 (PMC5513712; doi:10.1128/mBio.00494-17)
Supplement: TABLE S2 [file mbo003173376st2.pdf]

Table S2. Bacteria percent survival[a] in lungs and spleen of antibiotic-treated mice

		Time post-infection	
	Antibiotics	6 weeks	12 weeks
**Lungs**			
WT	RIF	100.2	4.2
Δ*phoY1*Δ*phoY2*	RIF	30.7	6.7
Δ*pstA1*	RIF	22.5	16.9
WT	INH	23.7	0.1
Δ*phoY1*Δ*phoY2*	INH	21.5	1.0
Δ*pstA1*	INH	23.9	0.6
**Spleen**			
WT	RIF	108.2	37.6
Δ*phoY1*Δ*phoY2*	RIF	57.5	2.9
Δ*pstA1*	RIF	38.1	8.7
WT	INH	23.0	0.1
Δ*phoY1*Δ*phoY2*	INH	3.7	0.2
Δ*pstA1*	INH	4.6	0.1

[a]Bacteria percent survival calculated as (strain CFU from drug-treated mice)/(strain CFU from untreated mice) x 100 at each time point.
