# Supplementary material for: Mycobacterium tuberculosis PhoY Proteins Promote Persister Formation by Mediating Pst/SenX3-RegX3 Phosphate Sensing
Source: mBio. 2017 Jul 11;8(4):e00494-17. doi: 10.1128/mBio.00494-17 (PMC5513712; doi:10.1128/mBio.00494-17)
Supplement: TABLE S3 [file mbo003173376st3.pdf]

Table S3. Oligonucleotide primers used for cloning, strain construction and real-time quantitative RT-PCR (qRT-PCR) in this study. Restriction enzyme sites used for cloning are underlined. Start and stop codons in primers used for construction of in-frame deletions are indicated in bold.

Name	Purpose	Sequence (5'-3')
dPY1F1	Upstream Δ*phoY1*	GATTAATTAAACGCACGCCGACATTGAC
dPY1R1	Upstream Δ*phoY1*	GACGCATGCCGTCCG**CAT**GGTGTCACG
dPY1F2	Downstream Δ*phoY1*	GACGCATGCTCCACTTAC**TGA**GAGGGTCTCG
dPY1R2	Downstream Δ*phoY1*	TCGGCGCGCCGTCAGCGGATCGTCGAACTC
dPY2F1	Upstream Δ*phoY2*	GATTAATTAACGCATCAAACGCCAGCAG
dPY2R1	Upstream Δ*phoY2*	GACGCATGCGGTCCG**CAT**GTGCTAAAGCCT
dPY2F2	Downstream Δ*phoY2*	GACGCATGC**TAG**GCCGAGCAAACATCCAC
dPY2R2	Downstream Δ*phoY2*	TCGGCGCGCCGGTTGGACCTCACGGACG
Y1F3	Check Δ*phoY1*	CATCCTGGGCACTGTCAACG
Y1R3	Check Δ*phoY1*	GGCTGACCATGTTGGCAACG
Y1seqF	Check Δ*phoY1*	CCGAGGTGCTCTCCCAG
Y1R4	Check Δ*phoY1*	CGATCCTCTGGCCGCAAC
Y2F3	Check Δ*phoY2*	GCCAGGTCACCACCGAGAG
dPTF2	Check Δ*phoY2*	GACGCATGCTTCGGCTAGGCCCGATG
PTF4	Check Δ*phoY2*	GGTCTCGGCCAGATGTTGAC
Y2R4	Check Δ*phoY2*	CGCTTACGGCATTTTCCAAGG
Y1-261F	Complement Δ*phoY1*	TTTCTAGAGACGACGTTTCGGCGGACAT
Y1-261R	Complement Δ*phoY1*	TTAAGCTTAACGATCCTCTGGCCGCAAC
Y2-261F	Complement Δ*phoY2*	TTTCTAGACCACCGTCAGCGTGCAGATAAG
Y2-261R	Complement Δ*phoY2*	TTAAGCTTCGGGTGGATGTTTGCTCG
QY1F1	Check complement Δ*phoY1*	GCCGGGATAGCGATGAAAC
QY1R1	Check complement Δ*phoY1*	CAGCGGCGCCAATGTC
QY2F1	Check complement Δ*phoY2*	CGAGCCCAAGTCGAAAAGG
QY2R1	Check complement Δ*phoY2*	CGGCCACCGGATGTTG
Y1PF	*phoY1* Southern	CGATGCGACTGCGGACCAG
Y1PR	*phoY1* Southern	GAGGGTCTCGTGTGGCGTTG
TY2PF	*phoY2* Southern	GGCTACTCGCAGGTGGTGTC
TY2PR	*phoY2* Southern	GACGGACACCTTGTTCAACCTC
sigAF5	qRT-PCR *sigA* forward	CTCAAACAGATCGGCAAGGT
sigAR5	qRT-PCR *sigA* reverse	CGCTAAGCTCGGTCATCAG
QY1F1	qRT-PCR *phoY1* forward	GCCGGGATAGCGATGAAAC
QY1R1	qRT-PCR *phoY1* reverse	CAGCGGCGCCAATGTC
QY2F1	qRT-PCR *phoY2* forward	CGAGCCCAAGTCGAAAAGG
QY2R1	qRT-PCR *phoY2* reverse	CGGCCACCGGATGTTG
QRXF1	qRT-PCR *regX3* forward	CAGCGTTCCGGTGATCATG
QRXR1	qRT-PCR *regX3* reverse	CAGGCCGACCACCTTGTC
Q0322F1	qRT-PCR *udgA* forward	TCAACCCCGACCGTATCGT
Q0322R1	qRT-PCR *udgA* reverse	CTCGCGGACGGCTACCT
Q0557F1	qRT-PCR *mgtA* forward	GCGGTCTACCAAACCGATGT
Q0557R1	qRT-PCR *mgtA* reverse	CGTGCTGTCATCGGAATGC
Q0784F2	qRT-PCR *rv0784* forward	CGATGTCGACGCGTTCTG
Q0784R2	qRT-PCR *rv0784* reverse	GAGCCACCAGCAACGATACC
ppk1-2 For	qRT-PCR *ppk1* forward	ATCGAAAACAGTCCCGTCAC
ppk1-2 Rev	qRT-PCR *ppk1* reverse	GCCGAGTCGTCTGGATACC

ppk2_For	qRT-PCR *ppk2* forward	CGTCTGGTGGTCATCTTCG
ppk2_Rev	qRT-PCR *ppk2* reverse	GGTTGAGGTACTCGGTGATCC
ppx1_For	qRT-PCR *ppx1* forward	ACGGTTGACCCGAGAGTG
ppx1_Rev	qRT-PCR *ppx1* reverse	TCCAGGACGGTCACACTG
ppx2_For	qRT-PCR *ppx2* forward	ACGTTGCAGGAGGTGTCC
ppx2_Rev	qRT-PCR *ppx2* reverse	CGGACAGTGTGGTCATCG
